# Evaluating potential impact of monoamine oxidase A missense L32S on the function of the enzyme monoamine oxidase A using *in silico* prediction tools and molecular modeling

**DOI:** 10.17912/micropub.biology.001414

**Published:** 2025-04-10

**Authors:** Jared Laughlin, Cynthia L Stenger, Hanna J Jefcoat

**Affiliations:** 1 Chemistry and Physics, University of North Alabama, Florence, Alabama, United States; 2 Mathematics, University of North Alabama, Florence, Alabama, United States; 3 Biology, University of North Alabama, Florence, Alabama, United States

## Abstract

Attention-deficit hyperactivity disorder (ADHD) is a neurodevelopmental disorder that affects 6-7% of people worldwide (Wilcutt, 2012). MAOA is a gene that encodes monoamine oxidase A, an enzyme responsible for the regulation and metabolism of monoamines thought to be associated with ADHD. This study investigates a leucine to serine swap at amino acid position 32 in FAD-binding domain of the enzyme monoamine oxidase A. Results from *in silico* prediction tools and molecular dynamics modeling provide evidence to support pathogenicity of the L32S missense variant of monoamine oxidase A.

**Figure 1. Characterization of L32S:**
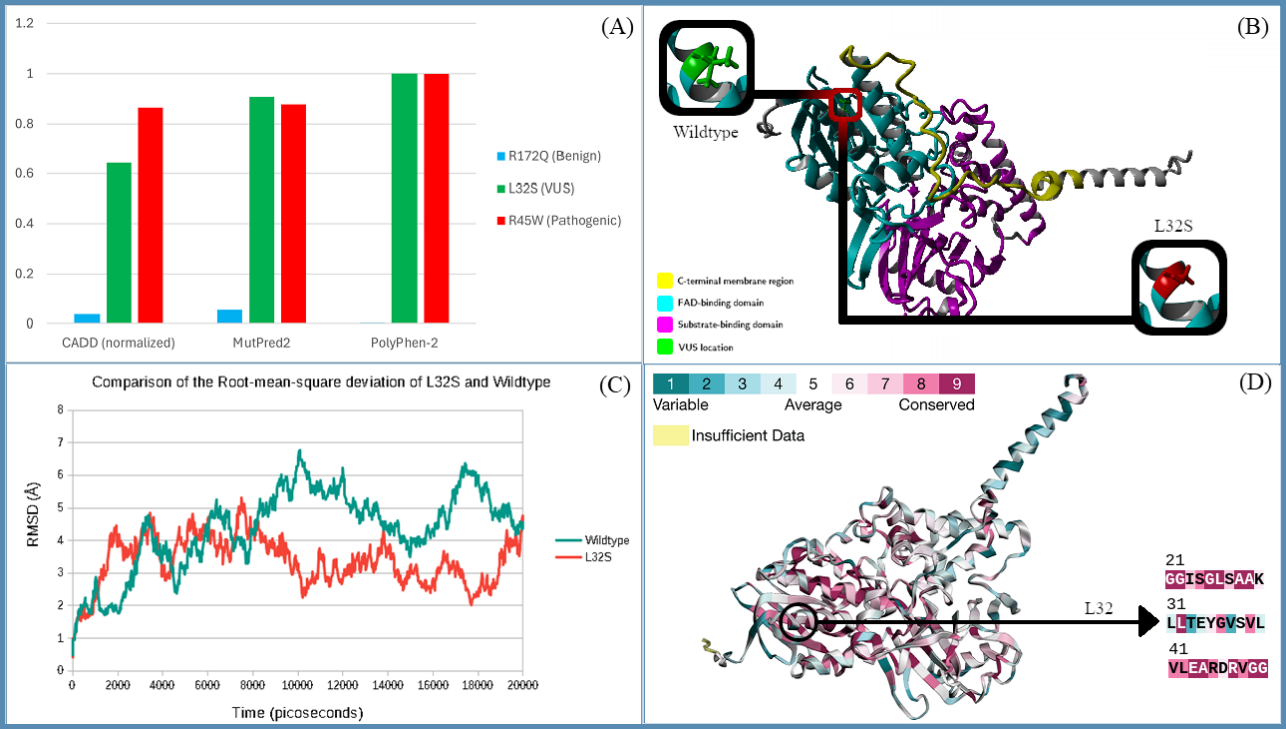
**A)** Analyzed pathogenicity using *in silico* predictors CADD, PolyPhen-2, and MutPred2 (Schubach et al., 2024; Adzhubei et al., 2010; Pejaver et al., 2020). All scores were normalized for comparison to show predicted pathogenicity of a known benign variant, the VUS being studied, and a known pathogenic variant. L32S is ranked as more highly pathogenic than the known pathogenic variant with all tools except CADD. **B)** YASARA homology modeling of monoamine oxidase A with highlighted swap. The legend indicates the functional domains of monoamine oxidase A. Note that L32 is located in the FAD-binding domain. **C)** Chart showing the root-mean-square deviation (RMSD) of L32S variant and wildtype monoamine oxidase A over a 20000 ps simulation. The first 10000 ps are considered equilibration and were not considered for analysis. (Gay et al., 2024) Note the stark divergence between the wildtype protein and the L32S variant **D)** ConSurf model of monoamine oxidase A (Yariv et al., 2023; Landau et al., 2005). The pink regions are more highly conserved than the blue regions. L32 is shown in a deep magenta color, indicating its high conservation grade of 9/9. The location of L32 on the protein is circled, with a segment of the conservation score table displayed on the right, showing the conservation scores of L32 and nearby residues 21-50.

## Description

Monoamine oxidase A catalyzes the oxidative deamination of monoamines. Monoamine oxidase A is expressed alongside monoamine oxidase B in most human tissues. It’s most abundant in the small intestine but is also expressed in the brain. Monoamine oxidase A is expressed in catecholaminergic neurons, where it metabolizes catecholamines such as norepinephrine and dopamine (Shih et al., 2004). Monoamine oxidase A catalyzes the first step in the processes of metabolism and excretion of catecholamines. Treatments for attention-deficit hyperactivity disorder (ADHD) target catecholamine systems in the brain, and ADHD is thought to be related to dysfunction in these catecholamine systems (Arnsten et al., 2011). Since monoamine oxidase A is expressed in these same neural circuits and impacts catecholamine concentrations in neurons, a variant in monoamine oxidase A could potentially contribute to the pathophysiology of ADHD.

One example of how monoamine oxidase A could contribute to the pathophysiology of ADHD is related to the high prevalence of substance use disorders (SUDs) in individuals with ADHD. Individuals with ADHD are much more likely to suffer from SUDs than non-ADHD individuals (Choi et al., 2022). SUDs are mediated by dopaminergic activity in the nucleus accumbens (Gardner, 2011). This is especially interesting as monoamine oxidase A is very highly expressed in the nucleus accumbens, where it degrades monoamines like dopamine (Kolla et al., 2020). Despite their reinforcing properties, catecholaminergic drugs used to treat ADHD reduce the risk of developing SUDs in individuals with ADHD and do not worsen existing SUDs (Hammerness et al., 2017; Levy et al., 2014; Wilens et al., 2003).

The leucine to serine swap at amino acid position 32 (L32S) has higher predicted pathogenicity scoring than both the selected benign and pathogenic variants for PolyPhen-2, and MutPred2, and L32S had a higher predicted pathogenicity score than the selected benign variant for CADD ( Figure 1A ) (Schubach et al., 2024; Adzhubei et al., 2010; Pejaver et al., 2020). The residue found in position 32 is very highly conserved according to the ConSurf server, with a conservation grade of 9/9 (Yariv et al., 2023; Landau et al., 2005). The residue is also located in the flavin adenine dinucleotide (FAD)-binding domain ( Figure 1B ). FAD is a redox active cofactor associated with monoamine oxidase A. The non-polar to polar swap from leucine to serine within the FAD-binding domain may alter the binding of this coenzyme to monoamine oxidase A, due to the abundance of polar bonds in the structure of FAD (National Center for Biotechnology Information, 2025). This variant also involves swapping a helix former, leucine, to a helix breaker, serine, on an alpha helix (Lewis, 1970). This can compromise the structural integrity of the protein and significantly alter the interaction between monoamine oxidase A and its environment. It is not currently known what structural effects this could have on the binding pocket of the enzyme. Although the swap occurred at position 32, the model shows consequential shifting throughout the sequence. The RMSD ( Figure 1C ) shows a stark divergence between the wildtype and the L32S variant.

## Methods

MAOA is one of the few genes known to be associated with metabolism of catecholamines. Monoamine oxidase A was chosen for study because of the wide variety of pharmaceutical drugs known to interact with this enzyme, opening up the possibility of future medical applications of this research. A variant of unknown significance (VUS) from MAOA L32S was chosen by comparing normalized scores from various pathogenicity predictors ( Figure 1A ). Using the ConSurf server, L32 was found to be highly evolutionarily conserved when compared to its homologues ( Figure 1D ) (Yariv et al., 2023; Landau et al., 2005). Slow homology models of the wildtype and variant proteins were constructed from a FASTA (P21397) sequence retrieved from UniProt. Using these homology models, molecular dynamics simulations were run using YASARA protein modeling to assess how the variant affects protein functionality. Molecular dynamics simulations model the movement of a protein in an aqueous environment over a period of 20000 picoseconds.
